# Engagement in Digital Mental Health Interventions: Can Monetary Incentives Help?

**DOI:** 10.3389/fpsyg.2021.746324

**Published:** 2021-11-18

**Authors:** Eliane M. Boucher, Haley E. Ward, Amelia C. Mounts, Acacia C. Parks

**Affiliations:** Happify Health, New York, NY, United States

**Keywords:** monetary incentives, digital interventions, mobile apps, behavior change, user engagement

## Abstract

Digital mental health interventions (DMHI) are scalable and cost-effective strategies for increasing access to mental health care; however, dropout rates associated with digital interventions are high, particularly for open-access digital interventions. While some studies have focused on predictors of dropout from digital mental health programs, few studies have focused on engagement features that might improve engagement. In this perspective article, we discuss whether monetary incentives (MI) are one avenue to increasing user engagement in DMHI. We begin by reviewing the literature on the effects of MI for behavior change in health domains (e.g., dietary behaviors, substance use, and medication adherence). Then, drawing on a pilot study we conducted to test the effects of different levels of MI on usage and improvement in subjective well-being among users of a DMHI (Happify), we discuss the potential applications of MI for DMHI, the potential drawbacks of financial incentives in this context, and open questions for future research.

## Introduction

In 2021, 85% of U.S. adults reported having smartphones and 77% reported having broadband Internet (Perrin, 2021). Given increasing access to smartphones and the Internet, along with difficulties meeting the demand for health services (Thomas et al., 2009; Osborn et al., 2016), demand for digital behavioral interventions is growing. Recent research estimates more than 10,000 behavioral health applications are available for download (Carlo et al., 2019). The subset of these interventions that are evidence-based and science-tested is a cost-effective way of disseminating treatments to large populations while reducing structural and attitudinal barriers associated with in-person treatment (see Fairburn and Patel, 2018, for a discussion). For instance, people view the lower time commitments (e.g., traveling time and waiting in provider’s office), increased flexibility (e.g., choosing the time of treatment and receiving treatment at home), and increased anonymity as perceived benefits of digital interventions (Gerhards et al., 2011; Ferwerda et al., 2013). People also seem willing to engage with health-related digital interventions; 50% of adults report using a fitness, medication-tracking, or other health-related application on their mobile phone (Mack, 2016). An even larger proportion (70%) reports being interested in using a smartphone application to monitor their mental health symptoms (Torous et al., 2014), particularly following the COVID-19 pandemic, when these tools were more accessible (Sorkin et al., 2021).

Internet-based interventions are also effective for addressing a variety of symptoms and conditions. Internet-based cognitive behavioral therapy (CBT) programs generally show moderate to large effects in terms of improving outcomes relative to control groups and may be as effective as face-to-face CBT (Cuijpers et al., 2008). Meta-analyses of digital interventions more broadly also find small to medium effects on outcomes including depression, anxiety, eating disorders (Sander et al., 2016), alcohol consumption (Kaner et al., 2017), and smoking cessation (Griffiths et al., 2018).

While the need for digital interventions is great, they have commonly struggled with low levels of engagement and high levels of dropout in clinical trials (Eysenbach, 2005) and in real-world roll-outs, especially when the cost to the user is minimal (Eysenbach, 2005; Cuijpers et al., 2008; Donkin et al., 2011). One review of user engagement for digital self-help interventions found that while downloads of such mobile applications are as high as 40,000 per month, only 7–42% of registrants engage in the moderate use of the application. Even fewer (0.5–28.6%) engage in sustained use or complete at least 6 weeks of the intervention (Fleming et al., 2018). Similarly, a review of real-world usage of mental health apps found that the median percentage of users who opened the app each day was 4%, and the median retention rate was 3.9% for 15 days and 3.3% for 30 days (Baumel et al., 2019). Consequently, while scalable and effective when used as recommended, low engagement prevents digital interventions from having their intended reach and impact.

Although some research has focused on the predictors of engagement at an individual level (Garnett et al., 2018), user-level variables, like personality and affect, may only account for a small portion of the variance in engagement (Sanatkar et al., 2021). Research on the impact of intervention features on engagement is largely in its infancy; however, what research exists suggests that programs involving more personalized advice and feedback (Strecher et al., 2008; Sharpe et al., 2017), a more individualized source (Strecher et al., 2008), email prompts (Alkhaldi et al., 2017), and text messages (Saslow et al., 2020) increase engagement. Research also suggests that support, from peers (Sharpe et al., 2017; Biagianti et al., 2018) or from therapists (Richards and Richardson, 2012; Mohr et al., 2013), increases engagement. Whereas monetary incentives (MI) are already used to promote user adherence to assessment schedules and data collection in research settings (Bentley and Thacker, 2004) and as a central intervention component in some key areas of behavior change, such as addiction (Petry, 2011), few studies have explored whether MI can increase engagement in digital interventions. Therefore, it is important to better understand what MI can (and cannot) do to improve engagement in digital mental health interventions (DMHI).

## Behavioral Economics and Engagement in Behavioral Interventions

Although there is scant research on the use of MI in digital interventions, such incentives have been used as a public policy tool to influence health behaviors through legislation and subsidies for centuries (Vlaev et al., 2019). More recently, the use of MI to reward healthy behaviors has grown in popularity with healthcare systems, insurers, and research organizations. This method of increasing healthy behaviors and decreasing problematic ones involves providing people with rewards contingent on reaching a predetermined goal (e.g., negative carbon monoxide tests for smoking cessation, number of steps reached, etc.) (Giles et al., 2014). These interventions typically offer cash rewards or vouchers that can be redeemed for goods or services (Sigmon and Patrick, 2012). The value of rewards typically varies (e.g., one meta-analysis found that payments for MI studies ranged from $5.16 to $786; Giles et al., 2014). Instead of rewarding individuals with outside funding, some programs utilize loss incentive manipulation requiring individuals to deposit cash at the onset, which is then refunded once a therapeutic goal is reached (Sykes-Muskett et al., 2015).

Contingency management (CM) was originally designed to treat substance abuse. Some CM programs exist as a stand-alone intervention and directly incentivize negative drug tests (Barnett et al., 2011), whereas others reward patients for adhering to treatment (i.e., psychotherapy or medication) or a combination of adherence and negative tests (Rosen et al., 2007). Research supports that CM effectively reduces cigarette, opioid, alcohol, marijuana, and benzodiazepine use (Petry, 2011). According to one meta-analysis, CM is the treatment with the greatest effect for substance use disorders (Dutra et al., 2008), and often has off-target effects on related psychiatric symptomatology (e.g., depression and anxiety; Miguel et al., 2017). Beyond substance abuse, CM has also been effective in improving medication and treatment adherence (Haff et al., 2015), vaccinations (Mantzari et al., 2015), fruit and vegetable intake (Gardiner and Bryan, 2017), and exercise (Washington et al., 2014), contributing to improved mental and physical health among the general population and individuals with chronic conditions (Vlaev et al., 2019) including severe mental illness, diabetes, obesity, HIV, tuberculosis, osteoarthritis, and hypertension (Ellis et al., 2021).

## Monetary Incentives in Digital Interventions: Some Preliminary Evidence

Despite evidence for the benefits of MI in face-to-face interventions, few digital interventions have incorporated these incentives; however, preliminary research suggests MI may be an effective engagement tool. In one study, participants downloaded a program called *Wellth*, which provides MI for engaging in specific health-related activities (e.g., medication adherence). Participants could earn up to $30 per month over 3 months. Of the 53 enrolled users, 54.72% completed the full 90-day program and 66% completed over 70% of the possible task check-ins *via* the app. Two-thirds of participants indicated MI increased their tendency to take their medication and adhere to their care plan (Granek et al., 2021). Similarly, in a naturalistic study of a 12-week digital fitness program, users who received MI for completing the program *via* their health plan were more than 12× more likely to complete the program than those who received no incentives (Wurst et al., 2020). However, to our knowledge, no randomized controlled trial on the effects of MI in digital interventions has been published.

Our research team conducted a pilot study on the impact of MI on engagement among Happify users that corroborates some of the observational evidence for MI. Happify is a self-guided DMHI accessible *via* smartphone or computer. It draws on various theoretical approaches to mental health including CBT (Beck, 1979), mindfulness-based stress reduction (Praissman, 2008), and positive psychology (Sin and Lyubomirsky, 2009), adapting evidence-based activities from these theoretical approaches into gamified versions. These gamified activities are organized into “tracks” that focus on a specific area of concern, like coping with negative thinking; users can also access activities outside tracks on demand. Happify use leads to significant improvement in mental health, including decreased symptoms of depression and anxiety, and increased resilience (Parks et al., 2018). Although engagement with the traditional, non-incentivized Happify program is better than comparable DMHI (Fleming et al., 2018), we were interested in whether MI could further improve engagement given that in some contexts, like clinical settings, higher levels of engagement may be required for clinically meaningful improvement.

In our study, new Happify users were randomly assigned to either a no incentives condition (*n* = 41), a sweepstakes condition where users were entered into prize drawings but received no MI (*n* = 41), or varying levels of MI (*n* = 181). Participants in MI conditions were rewarded for completing activities, progressing in tracks, and completing in-app assessments. Incentive conditions offered incrementally greater compensation for these tasks: base level (similar to what participants might earn in a research study; *n* = 21), 2× base level (*n* = 30), 4× base level (*n* = 66), 6× base level (*n* = 25), and 8× base level (*n* = 39). Aside from incentives, users had access to identical versions of the Happify commercial platform. We then tracked participants’ usage of the program over time and tested the impact of MI on usage and changes in well-being between a user’s first and last in-app assessment. Consequently, we focused on a subset of participants who completed at least two in-app assessments and thus were on the platform for 2 weeks or more.

Because the amount earned varied by user even within the same condition, we started by evaluating the relationship between MI and usage, treating the amount earned by each user as a continuous variable and time as a covariate (as this varied across users). Higher levels of compensation were associated with more usage, both in terms of active days on the platform, β = 0.93, *t*(260) = 38.78, *p* < 0.001, and activities completed, β = 0.92, *t*(260) = 38.70, *p* < 0.001. Higher levels of compensation earned also were associated with greater improvement in subjective well-being (based on our in-app assessment; see Parks et al., 2020, for a detailed description), β = 0.30, *t*(261) = 5.16, *p* < 0.001. Interestingly, the effect of MI on improvements in subjective well-being was mediated by the number of active days (Sobel = 2.22, SE = 0.08, *p* = 0.027), but not by the number of activities completed. This suggests that while MI increase both regularity and volume of usage, it is the increase in consistent usage that ultimately helps to improve outcomes associated with the intervention (in this case, well-being).

## Open Questions and Considerations

### What Amount of Incentive Is Optimal for Behavioral Change?

Although there is promising preliminary evidence for the benefits of MI in digital interventions, the amount required to encourage engagement is unclear. Observational research of the Canadian health app *Carrot*, which rewards users for completing health-related quizzes and health risk assessments, found that a 10% decrease in points awarded for completing a quiz was associated with a 1% decrease in quiz responses (Brower et al., 2020), suggesting the mere presence of MI, rather than the amount of the incentive itself, may be important.

**FIGURE 1:**
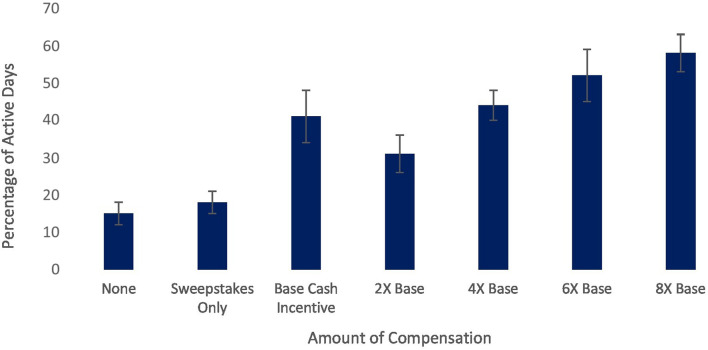
Relationship between cash incentive level and percentage of active days on a digital mental health platform.

**FIGURE 2:**
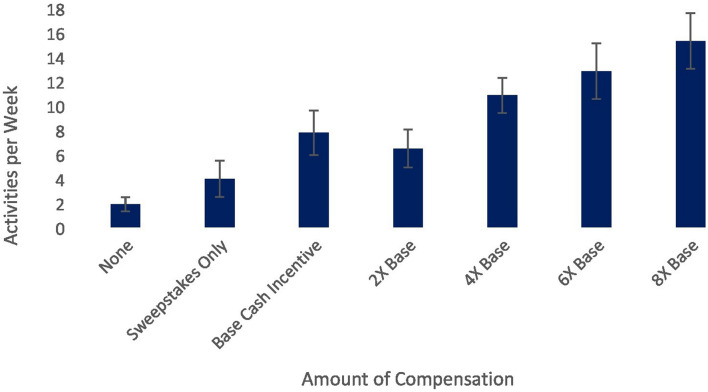
Relationship between cash incentive level and activities completed per week on a digital mental health platform.

Our pilot study also sheds some light on this issue. Although earlier we treated the total amount earned as a continuous variable, we can also separate participants based on the amount of incentive offered. In our pilot, MI resulted in a higher percentage of active days (relative to total days since signup) on the platform, *F*(6,256) = 11.45, *p* < 0.001, and activities completed per week, *F*(6,256) = 8.30, *p* < 0.001. Users who received no incentives engaged with the program roughly as recommended (approximately two to three activities per week; see Carpenter et al., 2016), accessing the platform on 15.44% of the days since signing up for Happify (see Figure 1), and completing an average of 1.90 activities per week (see Figure 2). Users in the sweepstakes only condition accessed the platform slightly more, but averaged more than twice the number of activities per week. MI appeared successful in boosting usage beyond the recommended level; users who were offered MI accessed Happify at least twice as often and completed at least twice the recommended number of activities per week. However, differences between the different incentive levels were small, and while the relationship appears mostly linear, the difference between the two lowest incentive levels is negligible, whereas subsequent increases seem more impactful. The error bars in Figures 1, 2 also illustrate the large variability between users in whether MI increase usage; thus, while only differences between the 2× and 8× base level groups were significant, the variability coupled with our small sample may have undermined our ability to detect differences between groups. Nevertheless, these data suggest that paying more will not necessarily yield better outcomes and increasing MI may have diminishing returns.

### Does Engagement and Behavior Change Revert When Incentives Are Removed?

How long MI should be offered and the impact of removing incentives on behavior also remain unclear. Some behavioral economists believe that increasing, rather than decreasing, incentives over time is most effective (Vlaev et al., 2019). This particular approach is common in programs for substance use; researchers argue that an escalating schedule of incentives should be offered until behavior change occurs, at which point incentives can be reduced without interfering with behavior (Stitzer and Petry, 2006). This monetary-based CM approach to substance use interventions has demonstrated maintained gains even after treatment termination (Davis et al., 2016). But in other domains, gains appear to fade once incentives are removed (Paloyo et al., 2015). For example, research using activity trackers suggests incentives help increase physical activity initially, but these effects are not sustained when incentives are removed (Finkelstein et al., 2016; Agarwal et al., 2021).

One reason for the loss in improvement when incentives are removed is that MI might only target extrinsic motivation, and inhibit the development of intrinsic motivation. Indeed, a commonly reported concern with implementing MI widely is that it may hinder patients’ development of intrinsic motivation to change health behaviors (e.g., Murayama et al., 2010). However, the effect of MI on intrinsic motivation remains unclear (Savioni et al., 2021), and evidence regarding the impact of financial incentives on intrinsic motivation is mixed (Petry et al., 2017). Relatedly, research on DMHI broadly, as well as specific research on the impact of MI (including our own pilot study) tend to operationally define engagement with usage metrics (e.g., active days and activities completed) and thus focus primarily on *technology engagement*. There is very little research that examines *patient engagement* in this context, or how MI impacts users’ emotional and cognitive states regarding the intervention, the health behavior, or their underlying condition. This is an important distinction because usage metrics, or technology engagement, may be indicative of passive adherence that is ineffective at promoting health management and quality of life long-term (Graffigna et al., 2014; Barello and Graffigna, 2015), which may also explain why improvements in engagement or behavioral outcomes associated with MI are not always sustained once MI are removed.

Currently, there is no research exploring the effects of MI on motivation, how MI affects people beyond their observable behavior and symptom change, or on reducing or removing MI within digital interventions, and few studies of in-person interventions include follow-up periods beyond 6 months (Giles et al., 2014). Testing different MI models (CM vs. reduction over time vs. constant), the impact of extinction, the effects of MI on motivation to change, and moving beyond technological engagement are important avenues for future research. These research questions will help resolve the issue of whether or not MI can lead to real, lasting change.

### Who Responds to Monetary Incentives and Who Does Not?

As discussed earlier, we noted a lot of variability in usage in our pilot study, even within conditions. The total amount earned also varied substantially within conditions (SDs ranging from $26.48 to $45.01), suggesting that even if people are offered larger incentives, they may not fully earn that incentive. This variability suggests some people may be more responsive to MI than others. For example, in substance use interventions, the impact of CM may depend on how responsive patients are to standard outpatient treatment (Forster et al., 2019). The benefits of MI may also depend on the behavior targeted by the intervention. Indeed, while CM appears effective for treating substance use, as noted earlier, the effects of MI on exercise may be temporary (Finkelstein et al., 2016; Hooker et al., 2018; Agarwal et al., 2021). Currently, MI research focuses on a single condition/behavior, so the extent to which effects vary depending on the condition or behavior of interest remains unclear. Research comparing MI effects across conditions will therefore be important to understand the contexts in which they are most effective.

Culture also plays a central role in how incentives are perceived; while they can be very effective if tailored to the target culture, a lack of cultural alignment can hinder success (Martin et al., 2017). For example, some research suggests smaller compensation amounts may be more effective for people from lower socioeconomic backgrounds (Vlaev et al., 2019). In addition, although motivation differs as a function of individualism and collectivism (Markus and Kitayama, 1991), research on the impact of MI in digital interventions, including our pilot study, included predominantly Western samples. Some research points to differences between Eastern and Western cultures in responses to MI on other behaviors, like purchasing intentions (Tercia and Teichert, 2016), emphasizing the importance of considering cultural differences. However, little research has focused on understanding whether individual or cultural differences influence the effectiveness of MI. To be scalable, it will be important to understand the factors that predict a patient’s response to MI in digital interventions, including personality and cultural background.

## Conclusion

Engagement is a crucial aspect of any DMHI (Eysenbach, 2005), making MI a hot topic in digital therapeutics. If we can determine how to optimize these incentives, there is great potential to increase uptake and retention metrics. Although the first digital therapeutic to receive FDA clearance, reSET, uses CM as part of its intervention (Maricich et al., 2021), there are still many questions we have yet to answer about the impact of MI in digital interventions. For example, do MI drive better clinical outcomes? Must participants always be rewarded to maintain usage? Is the cost/benefit ratio of the incentive and the relief of disease symptoms one that makes sense to payors? And in what cases are the effects driven by the incentives vs. true behavioral change that has taken place as a result of the incentives? Furthermore, there are ethical considerations and potential psychological implications of using money to incentivize participation (Ashcroft, 2011; Vlaev et al., 2019).

Our pilot data suggest MI have a clear impact on usage with our DMHI, but the impact is inconsistent. Therefore, the conclusion that “monetary incentives improve engagement” must be tempered by several caveats: (1) only on technological engagement or usage, (2) only if high usage levels are spread out over multiple sessions, (3) more money does not necessarily mean more benefit, (4) only if done in a way that leads to sustained change, and (5) there is large variability in how people respond to MI. Consequently, intervention developers must determine whether the additional cost of offering MI is worth the potential boost in engagement. For example, in the pilot we discussed here, participants in the highest incentive condition, who were offered 8× the amount as those at the lowest incentive level, were active an average of 12.50 more days and completed an average of 67.58 more activities. This increase may be worth the additional cost for some interventions, but not for others. Additional research adequately powered to explore the impact of incentive amount on both technological and patient engagement will be crucial to understanding how to optimally implement MI into digital interventions. It will take scientific input from every digital mental health manufacturer and science group using MI before we can fully understand the value, and potential pitfalls, of such incentives for improving digital product engagement. Nevertheless, we suggest that while a potentially useful tool, MI may not be a one-size-fits-all solution to the problem with engagement in digital interventions that one might have hoped for.

## Data Availability Statement

The data analyzed in this study are subject to the following licenses/restrictions: the data for the pilot study described in this article were collected as part of market research conducted by Happify Health using the commercial Happify platform. Due to user privacy concerns, individual data will not be shared and we present outcomes in aggregate form only. Interested readers may contact the corresponding author for additional information. Requests to access these datasets should be directed to EB, eliane@happify.com.

## Ethics Statement

Ethical review and approval was not required for the study on human participants in accordance with the local legislation and institutional requirements. Written informed consent for participation was not required for this study in accordance with the national legislation and the institutional requirements. Data discussed in this manuscript were collected as part of market research and covered by our user agreement.

## Author Contributions

EB and AP conceptualized the idea for this manuscript. EB analyzed the pilot data. EB, HW, and AP wrote the manuscript. AM provided manuscript support. All authors contributed to the article and approved the submitted version.

## Conflict of Interest

EB, HW, and AP are employees of Happify Health. AM was employed by Happify Health at the time of submission.

## Publisher’s Note

All claims expressed in this article are solely those of the authors and do not necessarily represent those of their affiliated organizations, or those of the publisher, the editors and the reviewers. Any product that may be evaluated in this article, or claim that may be made by its manufacturer, is not guaranteed or endorsed by the publisher.
